# Fluorescence-Based Quantitative and Spatial Analysis of Tumour Spheroids: A Proposed Tool to Predict Patient-Specific Therapy Response

**DOI:** 10.3389/fdgth.2021.668390

**Published:** 2021-05-28

**Authors:** Loredana Spoerri, Gency Gunasingh, Nikolas K. Haass

**Affiliations:** The University of Queensland Diamantina Institute, The University of Queensland, Brisbane, QLD, Australia

**Keywords:** tumour spheroid, tumour micro-environment, spatial analysis, intra-tumoural heterogeneity, anti-cancer drug response, hypoxia, cell death, immune cell infiltration

## Abstract

Tumour spheroids are widely used to pre-clinically assess anti-cancer treatments. They are an excellent compromise between the lack of microenvironment encountered in adherent cell culture conditions and the great complexity of *in vivo* animal models. Spheroids recapitulate intra-tumour microenvironment-driven heterogeneity, a pivotal aspect for therapy outcome that is, however, often overlooked. Likely due to their ease, most assays measure overall spheroid size and/or cell death as a readout. However, as different tumour cell subpopulations may show a different biology and therapy response, it is paramount to obtain information from these distinct regions within the spheroid. We describe here a methodology to quantitatively and spatially assess fluorescence-based microscopy spheroid images by semi-automated software-based analysis. This provides a fast assay that accounts for spatial biological differences that are driven by the tumour microenvironment. We outline the methodology using detection of hypoxia, cell death and PBMC infiltration as examples, and we propose this procedure as an exploratory approach to assist therapy response prediction for personalised medicine.

## Introduction

Cancer treatment can be primarily subdivided into two main approaches: targeted therapy, where cancer cells are killed directly by a cytotoxic drug, and immune therapy, where cancer cell death is achieved *via* reactivation of the patient's immune system. Each therapy type has its own advantages and disadvantages, but an important limitation they have in common is frequent drug insensitivity and disease relapse (1).

Primary and acquired resistance to targeted therapy depends on both cancer type and cytotoxic effector. In addition to the substantial development of acquired resistance, there is primary resistance. For instance, more than 40% BRCA1/2-deficient ovarian cancer patients fail to respond to PARP inhibitors from the start of the treatment (2). Conversely, while the vast majority of patients with BRAF-mutant melanoma has objective responses or temporary stable disease when treated with a BRAF inhibitor, they inevitably relapse, almost always in the first 1–2 years of therapy due to acquired resistance (3). Inter- and intra-tumoural heterogeneity are considered amongst the main culprits of resistance to cytotoxic targeted therapy.

Cancer immunotherapy has proven to have more durable response, with limited development of acquired resistance. However, the lower initial response (4) and often severe adverse effects (5) demand an improved treatment response prediction. Currently available biomarkers have limited predictive capacities leading to unsatisfactory clinical benefit in the majority of treated patients, and therefore to unforeseen significant side effects, financial costs, and health care burden (6).

Tumour spheroids provide a physiologically relevant tumour model to assess specific anti-cancer therapy outcome functionally and therefore independently from biomarkers (7–9). Compared to 2D culture, spheroids present a more comprehensive set of biological features that can impact therapy response. These include extracellular matrix surrounding the cells, compressive stress and a distribution of oxygen and nutrients following a gradient throughout the whole mass, resulting in hypoxia, cell cycle arrest and other localised biological alterations. Treatment sensitivity of distinct cell clusters is expected to be defined by the combination and resulting effect of these localised changes.

We describe here three methods based on quantitative and spatial fluorescence analysis of spheroids that may be used to assist treatment prediction. The first method serves to identify heterogeneous clusters within spheroids, more specifically distribution of populations of hypoxic cells (10, 11). The second method allows to spatially localise cell death within the spheroids (12, 13). The third method assesses immune cell infiltration into patient-derived spheroids. Beyond measuring the total number of infiltrating peripheral blood mononuclear cells (PBMCs), this technique presents the advantage to assess infiltration depth and spatial correlation with tumour heterogeneity. The real potential of these methodologies lies in the combination of these techniques to obtain insight on treatment response of specific heterogeneous cell populations.

Briefly, spheroids are generated using patient-derived melanoma cells based on a non-adherent surface method (7, 9, 14). Prior to harvest and fixation, spheroids are incubated with (1) Hypoxyprobe™-1 to detect hypoxia, (2) DRAQ7™ far-red emitting compound to assess cell death, or (3) patient-isolated and fluorescently labelled PBMCs to study immune cell infiltration. Spheroids are then prepared for fluorescence imaging. Image analysis consists in the measurement of the location of individual cells relative to the spheroid surface and to their target fluorescent signals, using the open-source image analysis software Fiji-ImageJ and CellProfiler. This information is then used to assess spatial and quantitative correlation amongst features of interest, for instance spatial distribution of cell death, hypoxic cell clusters and immune cell infiltration.

We regularly use these techniques in our laboratory to better understand molecular mechanisms in cancer (in particular melanoma) with the ultimate goal to improve therapy. However, we have not yet assessed their ability to predict treatment response in clinical settings. Based on the clinical relevance of spheroids as tumour models combined with the importance of tumour hypoxia, cell death and immune cell infiltration to predict therapy outcome, we propose here an explorative application of these techniques to assist the process of personalised medicine. For example, hypoxic cell populations within a tumour are typically less sensitive to therapy (15), however they present the advantage to be targetable with greater selectivity over healthy tissue by hypoxia-activated prodrugs (16). Nevertheless, the activity of these drugs appears to depend not only on hypoxia but also on the expression of specific prodrug-activating reductases and intrinsic cell sensitivity to the cytotoxic effector (17). Spheroids generated with patient-derived cells could be used to closely mimic the physiology of hypoxic cells within the patient's tumour(s) and assess whether treatment with hypoxia-activated prodrugs would effectively kill these populations of cells. Poor anti-tumour lymphocyte infiltration [e.g., cytotoxic T cells (CTL) or natural killer (NK) cells] is amongst the main reasons for immune checkpoint inhibitor resistance (18). Thus, immune cell infiltration and activation in response to immunotherapy with varying doses and timing could be assessed on patient-derived spheroids exposed to patient-derived PBMCs. This could assist the decision which inhibitor and which regimen a patient should receive.

## Materials and Equipment

### Spheroid Formation

1. Agarose
2. Sterile PBS
3. Microwave
4. Cell medium
5. 96-well flat bottom plate
6. Pipettes and tips
7. Hemocytometer or automated cell counter
8. CO_2_ incubator

### Pimonidazole Staining

1. Cell medium
2. Pimonidazole HCl
3. Microcentrifuge tubes
4. Pipettes and tips
5. CO_2_ incubator

### DRAQ7™ Staining

1. Cell medium
2. DRAQ7™
3. Microcentrifuge tubes
4. Pipettes and tips
5. CO_2_ incubator

### PBMC Staining

1. Patient's blood
2. Ficoll-Paque™
3. PBS
4. EDTA
5. CellTracker™ Deep Red Dye
6. FBS
7. Cell medium
8. 50 ml tubes
9. 15 ml tubes
10. Microcentrifuge tubes
11. Centrifuge
12. Microcentrifuge
13. Pipettes and tips
14. CO_2_ incubator

### Spheroid Fixation, Sucrose Cryo-Protection, and Cryosectioning

1. PFA
2. PBS
3. Sucrose
4. Curved-tip forceps
5. OCT
6. Casting moulds
7. Petri dish
8. Dry ice
9. Cryostat
10. Blades
11. Glass slides

### Spheroid Fixation, Sucrose Cryo-Protection, and Cryosectioning

1. Staining jars
2. RO water
3. Triton X-100
4. TBS
5. Liquid blocker
6. BSA
7. Anti-pimonidazole mouse IgG1 monoclonal antibody Hyproxyprobe-1 MAb1
8. Anti-mouse fluorescent antibody
9. DAPI
10. MOWIOL mounting medium
11. Coverslips

### Spheroid Clearing

1. Quadrol® [N,N,N′,N′-tetrakis(2-hydroxypropyl)ethylenediamine]
2. Urea
3. Triton X-100
4. Sucrose
5. NaCl
6. Chamber slides or low-profile glass bottom multi-well plates

### Imaging

1. Confocal microscope

### Software-Based Image Analysis

1. Fiji-ImageJ program
2. CellProfiler program

## Methods

### Part I—Lab-Based Work

#### Spheroid Formation

**Figure 1:**
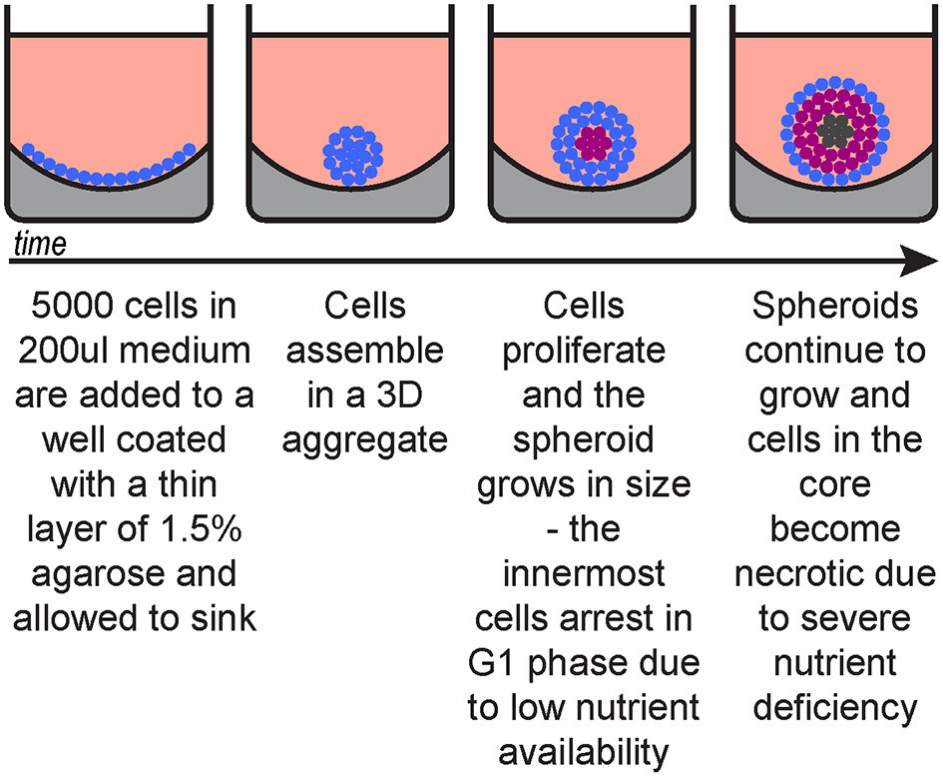
Spheroid generation timeline. Blue, cycling cells; magenta, G1-arrested cells; dark grey, necrotic cells.

This procedure describes spheroid generation using a liquid overlay technique (Figure 1) optimised for melanoma cell lines, however it can be substituted with other spheroid formation protocols suitable for the cells of interest, including patient-derived cells.

- Prepare 1.5% tissue culture agarose in sterile PBS.
- Microwave until agarose is fully dissolved.
- Immediately add 50 μl per well (96-well plate).
- Once set (~30 min) add 200 μl medium with 5,000 cells per well (2.5 × 10^4^/ml).
- Culture for 72 h in an incubator (37°C, 5% O_2_).

The detailed procedure for spheroid formation has been previously described by Spoerri et al. (14).

#### Fluorescence Staining

**Figure 2:**
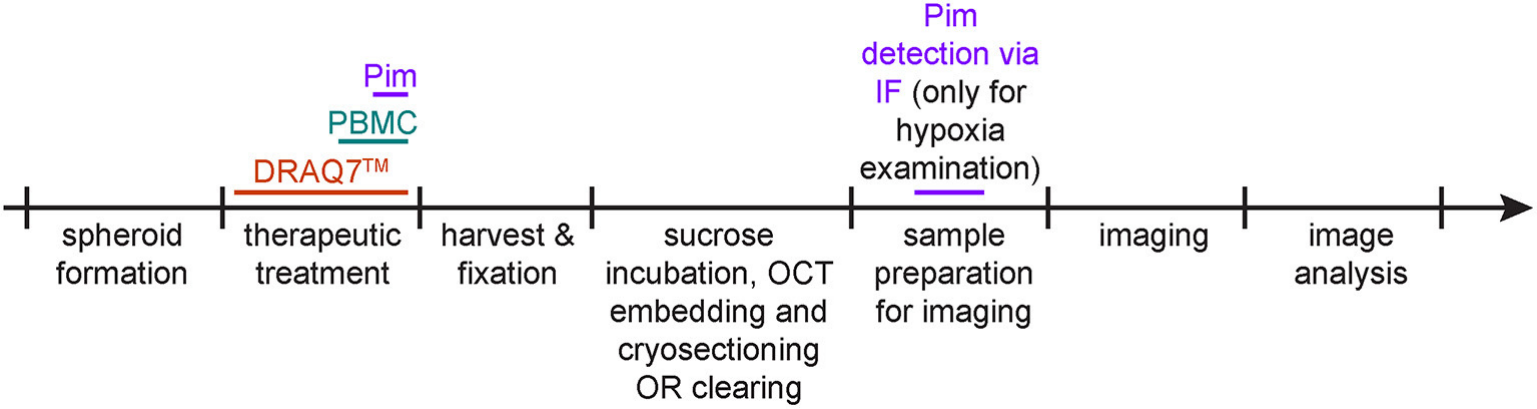
Protocol schematic. Spheroid treatment timeline for detection of hypoxia *via* pimonidazole staining (purple), of cell death *via* DRAQ7™ (orange) and of infiltrating PBMCs (aquamarine).

The following three procedures describe the steps for detection of hypoxia, cell death and infiltrating PBMCs within spheroids. They can be applied separately or in combination. Figure 2 depicts a timeline of when these procedures are applied (between spheroid formation and imaging).

If co-detection of hypoxia, cell death, and infiltrating PBMCs is desired in the same sample, consider how to achieve optimal spectral separation for imaging. DRAQ7™ and DAPI, respectively, emit far-red and blue fluorescence, thus consider detecting hypoxia and infiltrating PBMCs through green or red fluorescence.

##### Spheroid Incubation With Hypoxyprobe™-1 to Detect Hypoxia

This protocol describes pimonidazole detection with two Hypoxyprobe™ Kit reagents, (1) pimonidazole HCl and (2) anti-pimonidazole mouse IgG1 monoclonal antibody Hyproxyprobe-1 MAb1 in combination with a general goat anti-mouse IgG (H + L) highly cross-adsorbed secondary fluorescent antibody.

- Add pimonidazole to spheroid culture medium to a final concentration of 100 μM and incubate for 2 h under the same culture conditions (e.g., 37°, 5% CO_2_).
- Transfer spheroids to a clean microcentrifuge tube according to the experiment conditions (spheroids representing technical repeats can be pooled and transferred into the same tube, use a pipette with a wide bore tip to avoid damaging the spheroids).
- Allow spheroids to settle on the tube bottom by gravity (this should take less than a minute, depending on spheroid size, shape and density).
- Carefully remove the supernatant without aspirating any spheroid.
- Remove supernatant and wash once with PBS.

##### Spheroid Incubation With DRAQ7™ Far-Red Emitting Compound to Assess Cell Death

- Add DRAQ7™ to the spheroid culture medium to a final concentration of 3 μM.
  This can be done before spheroids form, i.e., adding DRAQ7™ to the single cell solution used to seed spheroids, or at a later stage once spheroids have already formed. In the latter case, allowing at least 24 h incubation will allow DRAQ7™ diffusion throughout the spheroids. We have observed (data not shown) that DRAQ™ fluorescence in spheroids remains clearly detectable up to 5 days post-addition to the culture medium.
- At the end of the incubation gently aspirate each spheroid along with some of the medium present in the well and transfer it to a fresh tube (use a pipette with a wide bore tip to avoid damaging the spheroids).
- Allow spheroids to sink by gravity then remove as much medium as possible without aspirating any spheroid.
- Wash once with PBS.

##### Spheroid Incubation With Patient-Isolated and Fluorescently Labelled PBMCs to Study PBMC Infiltration

This procedure describes staining of PBMCs with CellTracker™ Deep Red Dye, but other non-toxic fluorescent cell stains may be used instead (optimisation of concentrations and incubation times may be required).

If detection of hypoxia and cell death in the same sample is desired, consider detecting infiltrating PBMCs through green or red fluorescence.

- Isolate PBMCs from a patient's blood sample of ~10 ml (e.g., using Ficoll-Paque™) and count cells.
- Resuspend PBMCs in 1 ml PBS with 10 μM EDTA (no FBS).
- Add 1 μl of CellTracker™ Deep Red Dye (add it to the side of the tube to allow dilution before cell contact) and incubate for 20 min at RT (in the dark).
- Stop the staining reaction by adding 5 ml PBS with 10% FBS.
- Centrifuge the tube at 500 × g (RCF) for 5 min.
- Remove supernatant and wash once with PBS with 10 μM EDTA.
- Resuspend in medium (same as the one used for spheroid culture but without antibiotic selection). Based on previous cell count (step 1) resuspend to a slightly higher concentration than 10 million/ml.
- Count PBMC again and if necessary, adjust volume to obtain a 10 million/ml concentration (quality control step).
- Take spheroids from incubator and remove 100 μl per well of existing culture medium.
- Add 1 million PBMC (100 μl of PBMC solution of 10 million/ml) per well and return to incubator for as long as required.
- At the end of the incubation gently aspirate each spheroid along with some of the medium present in the well and transfer it to a fresh tube (use a pipette with a wide bore tip to avoid damaging the spheroids).
- Allow spheroids to sink by gravity then remove as much medium as possible without aspirating any spheroid.
- Wash three times with medium then once with PBS.

#### Spheroid Harvest and Fixation

Spheroid should now be in PBS in a microcentrifuge tube following any of the three staining protocols described above.

- Allow spheroids to sink by gravity then remove as much supernatant as possible without aspirating any spheroid.
- Add warm (37°C) 4% PFA (in PBS) and incubate for 20 min at 37°C.
- Remove PFA, add PBS, allow spheroids to sink again, and remove the supernatant as done in step 2.

#### Sample Preparation for Fluorescence Imaging

Two main methodologies through which we prepare spheroids for imaging are described here. One is spheroid cryosection and the other is spheroid clearing. Spheroid cryosectioning can be time-consuming but provides thin spheroid slices that can be imaged with standard wide-field fluorescence microscopes. Optical clearing of spheroids reduces sample preparation time but requires imaging with confocal microscopes and may limit immunofluorescence staining as antibody diffusion into spheroids may not be homogeneous. Imaging of live, uncleared spheroids may be necessary for time-lapse based investigations. Acceptable image quality can be achieved for low-scattering spheroids (e.g., C8161 melanoma spheroids) or with the help of more specialised microscopy equipment, such as selected plane illumination microscopy (SPIM). This type of imaging requires the use of cell-expressed fluorescent proteins or exogenously added fluorescent compounds that are non-toxic and can permeate the spheroids.

##### Spheroid Cryosections

This methodology is recommended for antibody-based fluorescence staining, such as the procedure for hypoxia assessment whereby pimonidazole is detected *via* immunofluorescence.

*Sucrose Incubation, OCT Embedding and Cryosectioning*

- Incubate in 30% sucrose (in PBS) at RT until spheroids sink (they will initially float once resuspended in 30% sucrose). This should take about 1–3 h and the spheroids should now appear slightly more translucent. This step is necessary for cryoprotection.
- Gently aspirate the spheroids along with some of the supernatant and transfer them to a flat surface, e.g., the lid of a Petri dish (do not use the dish itself if its surface is culture-treated as the spheroids may stick to it, use a pipette with a wide bore tip to avoid damaging the spheroids).
- Add a layer of OCT at the bottom of a small casting mould, e.g., 1 × 1 cm.
- Use curved-tip forceps to pick a small amount of OCT and use it to transfer spheroids (use the OCT in between forceps tips as a spoon, spheroids tend to adhere to OCT, OCT provides a soft and therefore non-damaging support). Add spheroids (one by one) on top of the OCT layer that is in the casting mould by. Try to place the spheroids laying on their larger surface (spheroids, as the name indicates, are not spherical but spheroidal), this will help to stabilise them during OCT freezing and their orientation will be known when sectioning them. If desired, add multiple spheroids per casting mould.
- Place the mould on dry ice for a few minutes until OCT has solidified.
- Bring the mould back to RT and allow just enough time for the OCT surface to become slightly translucent again, then add a layer of OCT to cover the spheroids. This step will allow proper “sealing” between the two OCT layers to avoid them breaking apart during sectioning.
- Place the mould on dry ice for a few minutes until OCT has solidified.
- Using a cryostat-sectioned spheroid at a thickness of 20 μm. If a small mould was used for casting the OCT blocks, multiple sections can be placed per slide. Use glass slide with enhanced specimen-to-slide adhesion to avoid section detachment during processing. Adjust the cryostat temperature between −20 and −10°C according to the brand and type of OCT used. Too cold temperature will result in brittle sections that tends to break while too warm temperature will cause the sections to wrinkle.

*OCT Removal and Immunofluorescence* All volumes in this procedure can be gently added on top of the sections using a pipette. Volumes depend on the size of the contained environment created with the liquid blocker. Add just enough volume to ensure coverage of the sections during the whole incubation, ~5–15 μl per 10 mm^2^. A too small volume will dry out while a too large volume will break the liquid blocker barriers and leak out also resulting in drying out. Use of a humidified chamber is recommended to minimise evaporation during incubations. Agitation such as rocking or shaking is discouraged as it will increase the number of sections detaching from the slides.

- Remove OCT from slides by immersing the slides in staining jars containing RO water and incubate without agitation for 3 min at RT. PBS can be used instead of water but it will leave residual salts when drying.
- Carefully remove the slides from the jars to minimise detachment of spheroid sections and briefly place the slides resting vertically on paper to allow elimination of excess water.
- To permeabilise the sections, place the slides in a horizontal position and add 0.5% Triton X-100 (in TBS). Incubate for 8 min at RT. Slide immersion in staining jars after OCT removal and for all the following steps is discouraged as it will likely increase the number of detaching sections.
- Aspirate the permeabilisation buffer then add some water to rinse off any residual Triton X-100.
- Briefly place the slides resting vertically on paper to allow elimination of excess water.
  **For detection of hypoxia, continue to the next step (in that case, spheroids must have been incubated with pimonidazole during spheroid culture/treatment). Otherwise follow the protocol from step 12**.
- Using a liquid blocker (hydrophobic pen), draw around the sections to create contained environments for incubations. Separate environments according to different staining conditions, if more than one used. This allows to reduce incubation volumes which is convenient when using more expensive materials such as antibodies.
- To block, add 5% BSA (in TBS) to the sections and incubate for 1–2 h at RT.
- Add the anti-pimonidazole mouse IgG1 monoclonal antibody Hyproxyprobe-1 MAb1 (1/200 in blocking solution) and incubate overnight at 4°C.
- To wash, aspirate the primary antibody and replace with TBS. Incubate for 10 min at RT. Repeat twice.
- Add the anti-mouse fluorescent antibody (1/500 in blocking solution) and incubate for 1–2 h at RT.
- Repeat washing as described in step 9 (3× washes of 10 min each).
- Add DAPI (or any other DNA stain) in TBS and incubate for 10–15 min at RT, then perform 3 TBS washes as described in step 9. DAPI incubation can be incorporated with secondary antibody staining or in the last washing steps if immunofluorescence (e.g., for pimonidazole detection) is performed.
- Add a small drop of MOWIOL mounting medium to each section and apply a coverslip (No. 1.5) on top. Allow to harden before imaging. Seal with nail polish if using a mounting medium that does not harden.

##### Cleared Spheroid

*Optical Clearing* Whole spheroids can be imaged in confocal microscopy by clearing and mounting them in high refractive index mounting solution. This protocol describes modified CUBIC clearing for spheroids.

- Prepare reagent 1 by mixing Quadrol® [5% (w/w)], Urea [10% (w/w)], Triton X-100 [10% (w/w)], and NaCl [25 mM] in MilliQ water. (Note: Weigh Quadrol® first and then adjust weight of the other ingredients to obtain desired percentages.)
- Prepare reagent 2 by mixing Quadrol® [9% (w/w)], Urea [22% (w/w)], Triton X-100 [0.1% (w/w)], and Sucrose [44% (w/w)] in MilliQ water.
- Transfer fixed spheroids to 1:1 reagent 1 to PBS solution and incubate at 37°C for 1 h on a rotor.
- Replace solution with 100% reagent 1 and incubate at 37°C for 1–4 h, until the spheroids appear translucent all the way through.
- Mount spheroids in reagent 2 in chamber slides or low-profile glass bottom multi-well plate and let the spheroids equilibrate for 30 min before imaging. (Note: Spheroids should be completely submerged in reagent 2 while mounting. Incomplete submersion can lead to loss of signal while imaging as well as physical compression of spheroid due to the surface tension of the viscous mounting solution).

*Nuclei Staining*

Spheroids can be stained after reagent 1 treatment.

- For DAPI staining, transfer spheroids to PBS and add 0.5 μg/ml of DAPI and incubate for 1–2 h at room temperature on a rotor. The duration depends on the size and permeability of the spheroids.
- Wash spheroids in PBS in generous volume of PBS for 15 min on rotor, 3 times.
- Proceed to mounting spheroids in reagent 2 and imaging.

#### Imaging

This protocol describes the steps for imaging spheroid sections fluorescently stained for detection of hypoxia, cell death and infiltrating PBMCs. Imaging using a confocal microscope is recommended to achieve image quality allowing accurate single cell analysis.

- Set the microscope to a magnification that allows visualisation of the whole spheroid. If necessary, combine higher magnification with image stitching.
- Use the DAPI laser line (405 nm) to visualise the cell nuclei, centre the spheroid in the field of view and adjust the z variable to achieve maximal focus.
- Acquire DAPI signal image.
- Without changing the x and y variables switch to the laser line to visualise the pimonidazole/hypoxia staining (488 nm if a green fluorescent dye was used, 561 nm if a red fluorescent dye was used). Use this line to assess whether any minimal z variable change is required for optimal focus—this may be required to achieve optimal focus as the initial focus was established on a nuclear staining while pimonidazole staining is primarily cytoplasmic.
- Acquire pimonidazole/hypoxia signal image.
- Repeat step 4–5 to image infiltrating PBMCs (488 nm or 561 nm laser depending on the dye used to stain the PBMCs).
- Repeat step 4–5 to image dead cells (647 nm laser).

### Part II—Software-Based Image Analysis

#### Fiji-ImageJ Analysis

Basic knowledge of Fiji-ImageJ and CellProfiler software is required to understand and implement the following protocols. For this, both programs feature free web access to manuals and numerous forum discussions.

##### Batch Convert Microscopy Proprietary Files to TIFF Stack Files Using Fiji-ImageJ

The following protocol describes how to batch generate TIFF stack files containing multiple channels from microscopy proprietary files. A microscopy proprietary file contains all channels used for that file acquisition.

- Place all microscopy proprietary files to convert in a folder (containing no other files or folders).
- In Fiji-ImageJ run the following macro.
  *(Modified from:* *http://microscopynotes.com/imagej/macros/Convert_ND2_to_tif_whole_folder.txt**)*
  The command in line 3 opens a “Choose a Directory” window where you need to specify the folder where the microscopy proprietary files are located.
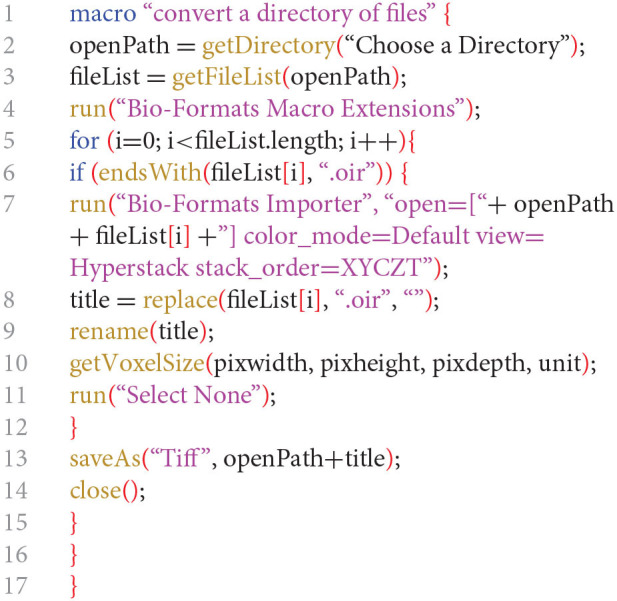
- A TIFF stack file is created for each microscopy proprietary file in the same folder.
- Move the TIFF stack files to a new folder (this will avoid microscopy files to be processed in the next step).

##### Batch Generate Single Channel Images From Stack Files Using Fiji-ImageJ

The following protocol describes how to batch convert TIFF stack files into TIFF single channel grayscale images.

- In Fiji-ImageJ run the following macro.
  *(Modified from:* *http://imagej.1557.x6.nabble.com/Batch-stack-to-images-with-renaming-td5011012.html**)*
  If <4 channels are present per microscopy file, the j variable in line 9 needs to be changed accordingly (i.e., ≤3 for 3 channels, ≤2 if 2 channels). Similarly, lines 13 and 14 may need to be deleted.
  Values assigned to variables in lines 12, 13, 14, and 15 define the suffixes of the generated TIFF single channel files and can be modified as desired.
  The command in line 2 opens a “Choose a Directory” window where you need to specify the folder where the TIFF stack files are located.
  The command in line 4 opens a “Choose a Directory” window where you need to specify the folder where you want to save the TIFF single channel files.
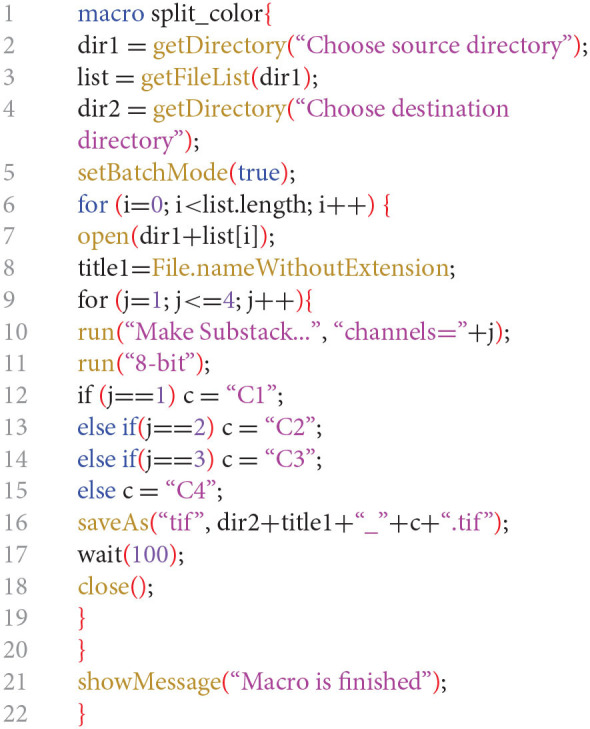
- TIFF single channel files are created in the selected destination folder.

#### CellProfiler Analysis

##### CellProfiler Analysis of Pimonidazole Staining

**Figure 3:**
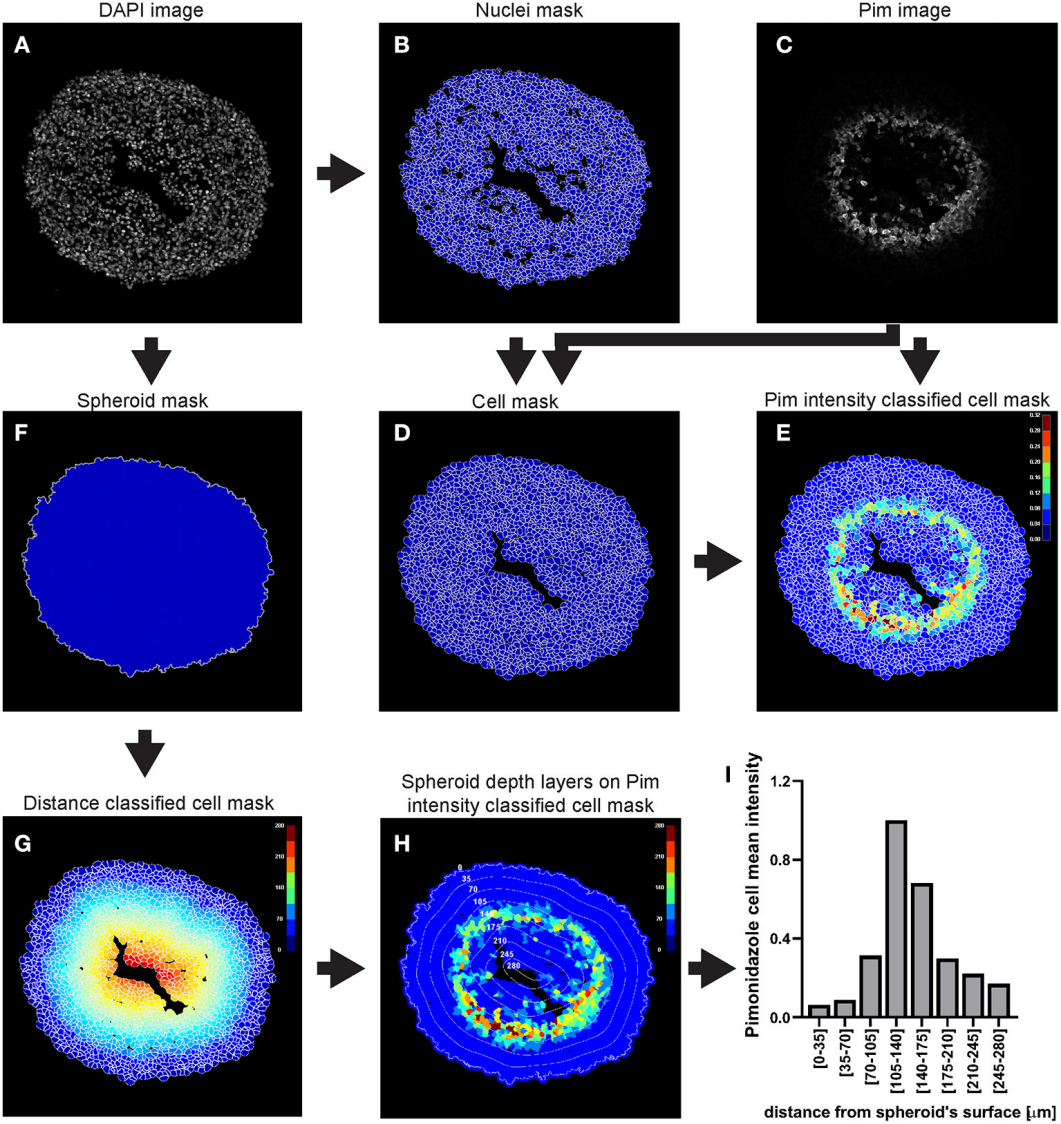
Main steps of CellProfiler pimonidazole image analysis. The DAPI image **(A)** is used to identify the nuclei a as primary objects **(B)** which in turn are exploited as “seed” regions, together with the pimonidazole image **(C)**, to outline their cytoplasmic area and define cells (nucleus + cytoplasm) **(D)**. Pimonidazole fluorescent signal is then measured for each cell **(E)**. The DAPI image **(A)** is also used to determine the spheroid outline **(F)** which is then exploited to measure the distance of each cell from the spheroid surface **(G)**. The data obtained from the CellProfiler analysis can be used to generate a profile of the pimonidazole cells mean intensity at different depths in the spheroid **(H,I)**. The colour-coded scales indicate signal intensity in arbitrary units **(E,H)** and distance in μm **(G)**. In **(H)** the numbers labelling the depth curves indicate the distance from the spheroid's surface in μm.

This example of analysis pipeline uses images fron a central cryosection of a spheroid that was generated with C8161 melanoma cells and incubated with 100 μM pimonidazole for 2 h, fixed, cryosectioned and then immunofluorescently stained (Figure 3).

**Figure 4:**
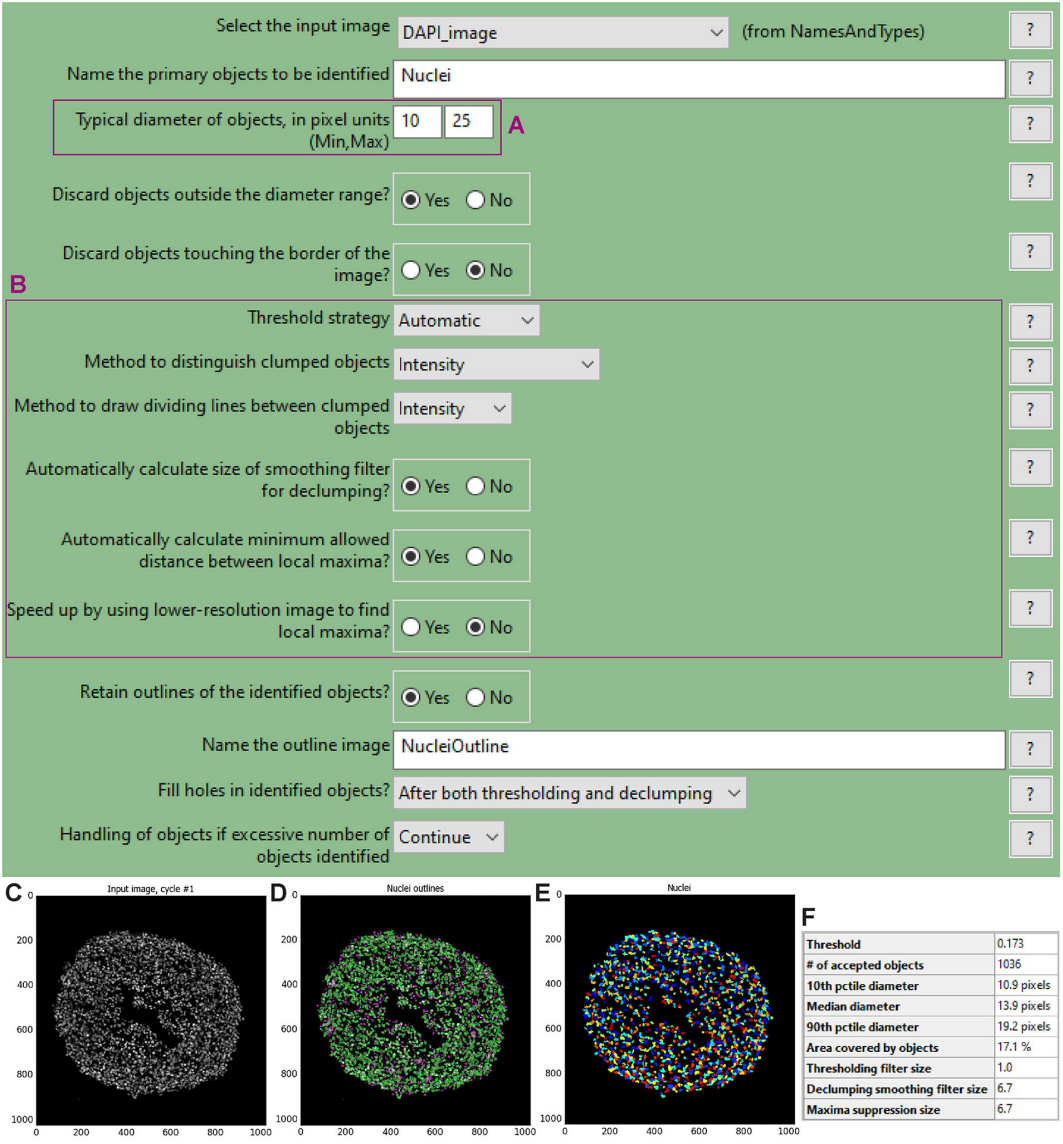
“IdentifyPrimaryObjects” module window and test mode results. The “IdentifyPrimaryObjects” module interface shows the parameters that can be adjusted to modify object identification accuracy such as the objects diameter range **(A)**. Other parameters include the threshold strategy, which affects the decision of whether each pixel will be considered a region of interest or background, and segmentation *via* the method to distinguish clumped objects and *via* the minimum allowed distance between local maxima **(B)**. For each steps of the pipeline a window displays the raw, original image **(C)**, the raw image overlaid with the coloured outlines of the identified objects and **(D)** the identified objects shown as a colour image where connected pixels that belong to the same object are assigned the same colour. For image **(D)** each object is assigned one of three colours: green for acceptable objects that passed all criteria, magenta for objects discarded based on size and yellow for objects discarded due to touching the image border. For image **(E)** the assigned colours are arbitrary. The windows also show a table with some of the settings selected by the user, as well as those calculated by the module in order to produce the objects shown **(F)**.

To choose the optimal parameters of each module use the test mode (“Start Test Mode”) which allows to run the pipeline step by step and to rapidly optimise the analysis parameters. Use the eye icon beside each module to immediately visualise after each step the results of that module (icon must show an open eye). An example of this process is explained for step a. of the following procedure and depicted in Figures 4C–F.

**a. Use the “IdentifyPrimaryObjects” module to identify the cell nuclei in the image**.

**Figure 5:**
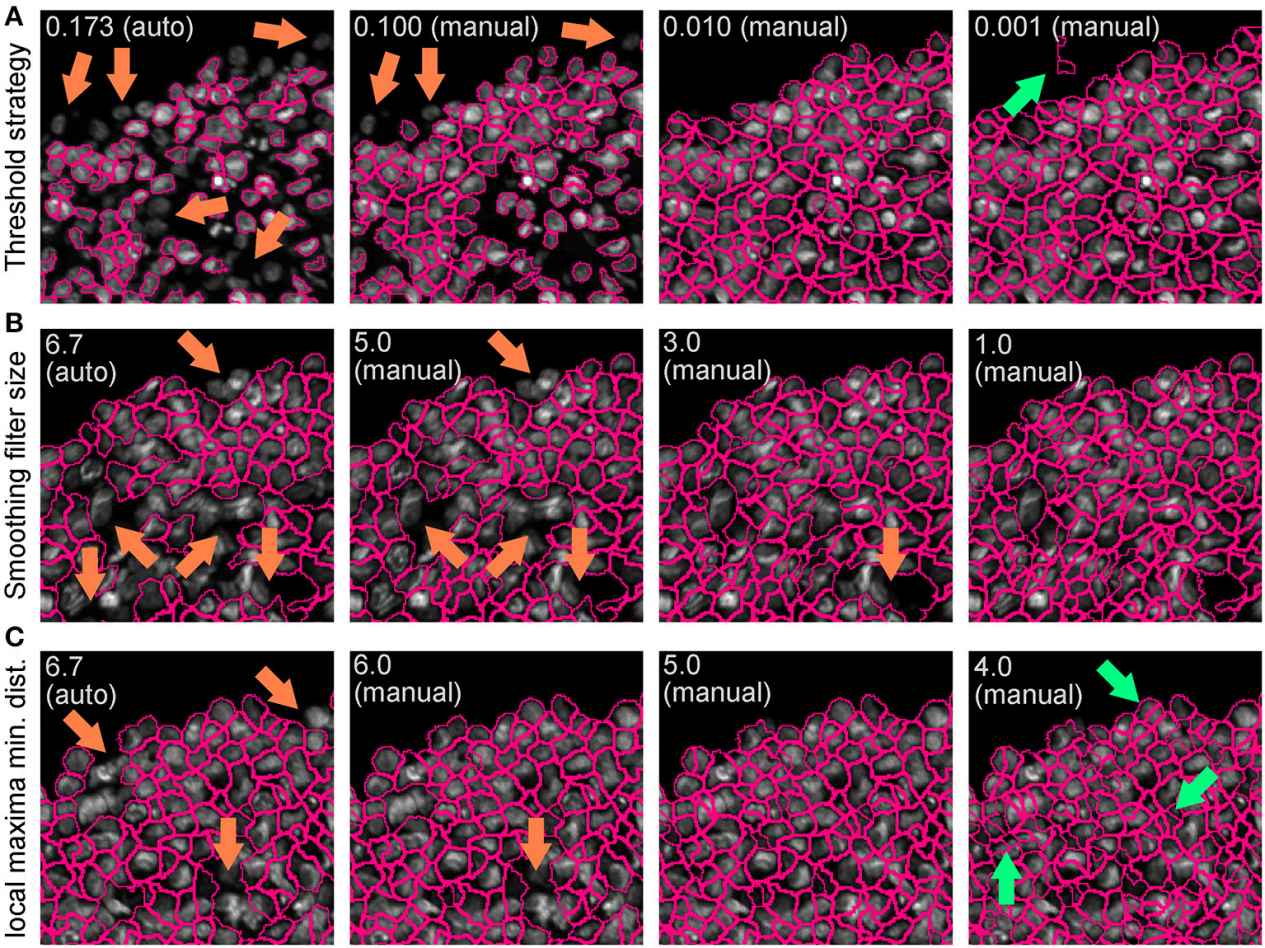
Effect of “IdentifyPrimaryObjects” parameters on object identification accuracy. Spheroid nuclei identification results from automatically (auto) and manually (manual) set threshold strategy **(A)**, size of smoothing filter for declumping **(B)** and minimum allowed distance between local maxima **(C)**. Magenta outlines indicate identified objects. Orange and green arrows indicate false negative and false object identification, respectively.

**Figure 6:**
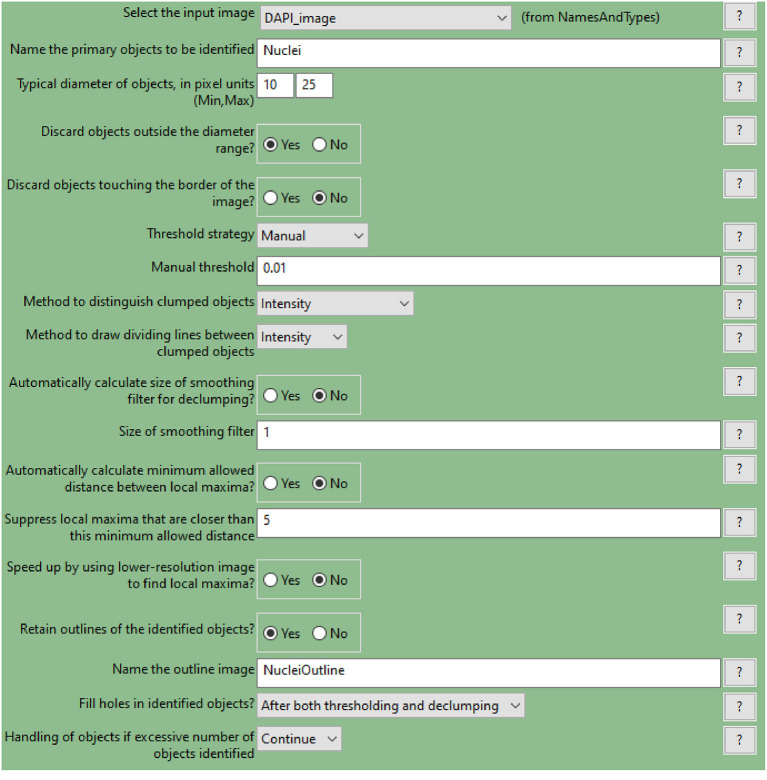
CellProfiler “IdentifyPrimaryObjects” module interface. Parameters used to identify spheroid nuclei for analysis shown in Figure 3.

- Use the nuclei image, in this example the DAPI image, as input.
- Indicate an estimate of the average diameter of the nuclei (Figure 4A) to help their identification as primary objects. This can be done by applying the Fiji-ImageJ measurement line tool to a few nuclei in the image.
- To begin, set the threshold strategy, the size of the smoothing filter for declumping and the minimum allowed distance between local maxima to be detected automatically (Figure 4B), then run this module as a single step in test mode. A new pop-up window will show the input image (Figure 4C), the nuclei outline image (Figure 4D), the image with only detected nuclei (Figure 4E) and the table of the automatically calculated settings (Figure 4F).
- Based on the obtained detection accuracy (Figure 4D), modify the automatically calculated settings (Figure 4F) and run the module again to observe how the detection accuracy has changed. Each object is assigned one of three colours: green (acceptable; passed all criteria), magenta (discarded based on size) and yellow (discarded due to touching the image border).
- Repeat this step until optimal detection is obtained. Figure 5 shows the effects of modifying the threshold strategy, the size of smoothing filter for declumping and the minimum allowed distance between local maxima on detection accuracy. Figure 6 indicates the settings used for the analysis shown in Figure 3.
- Depending on the image, automatic settings may produce accurate results without the need to adjust them.

**b. Use the “IdentifySecondaryObjects” to determine the cell outline (cytoplasm included)**

**Figure 7:**
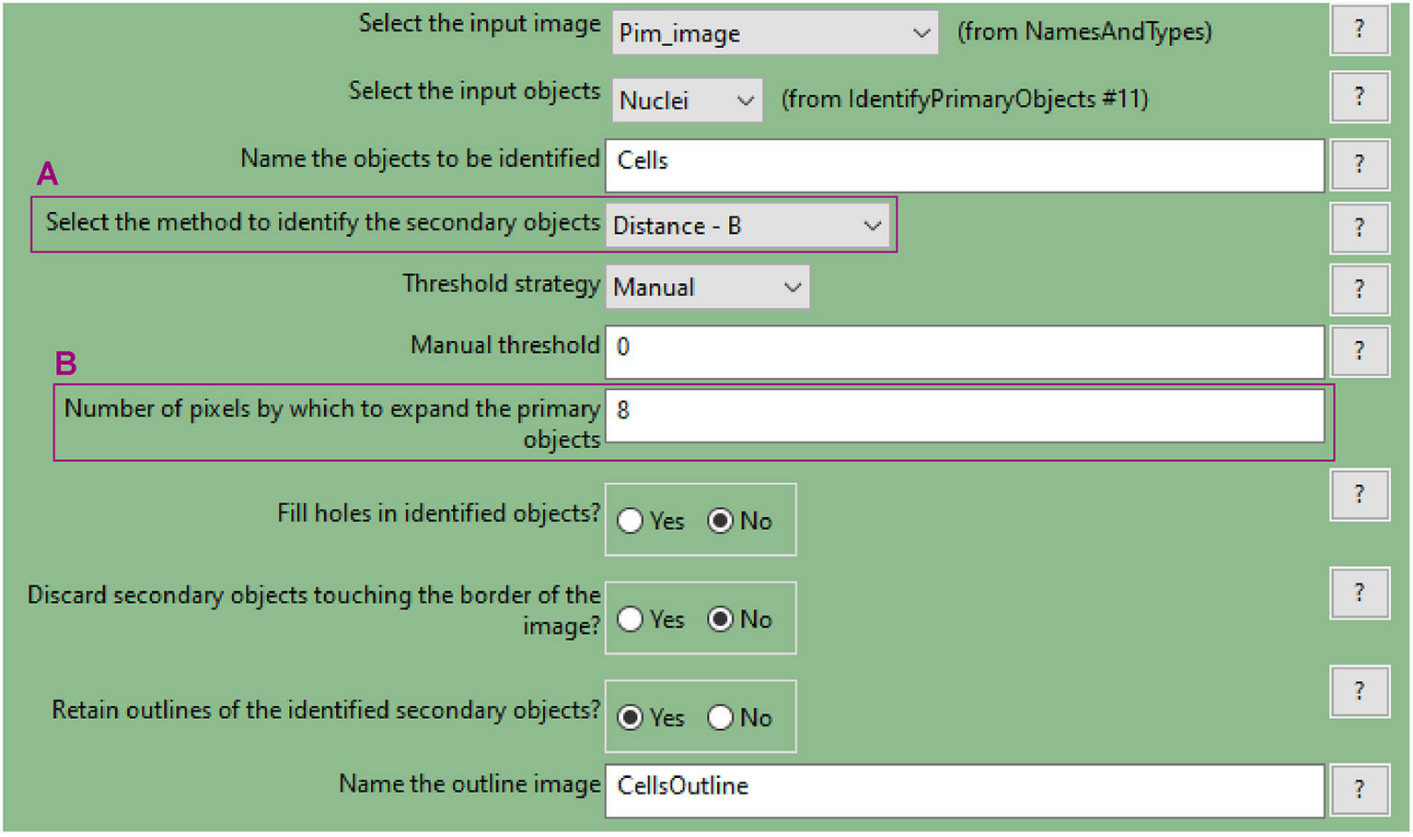
CellProfiler “IdentifySecondaryObjects” module interface. Parameters used to identify spheroid cells (cytoplasm included) for analysis shown in Figure 3.

- This module identifies cells (as defined by the outline of their cytoplasm) by using the nuclei as a “seed” region, then growing outwards until stopped by the image threshold or by a neighbour cell.
- Use the pimonidazole image as input. In this example, where the “Distance-B” method is applied (Figure 7A), the pimonidazole image is used to find the edges of the secondary objects for each nucleus. However, where pimonidazole signal is very low (cells in the spheroid periphery) the cell edges will be defined by the indicated number of pixels by which to expand the primary objects (Figure 7B).
- Use the test module to optimise accurate detection.

**c. Use the “MeasureObjectIntensity” module to quantify each individual cell's pimonidazole fluorescence**

**Figure 8:**
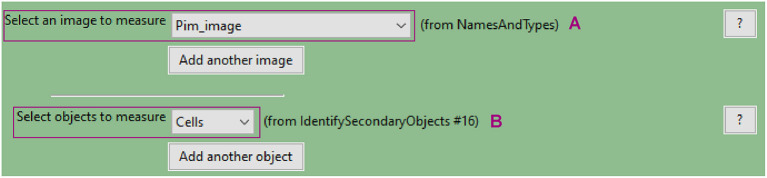
CellProfiler “MeasureObjectIntensity” module interface. Parameters used to measure cell pimonidazole fluorescence signal for analysis shown in Figure 3.

- This module extracts intensity features for each object based on one or more corresponding grayscale images. Measurements are recorded for each object.
- Intensity features include mean, median and integrated intensity amongst several others (refer to CellProfiler manual for details).
- Select the grayscale image (Figure 8A) and objects (Figure 8B) whose intensity you want to measure.

**d. Use the “IdentifyPrimaryObjects” module to identify the spheroid surface**

**Figure 9:**
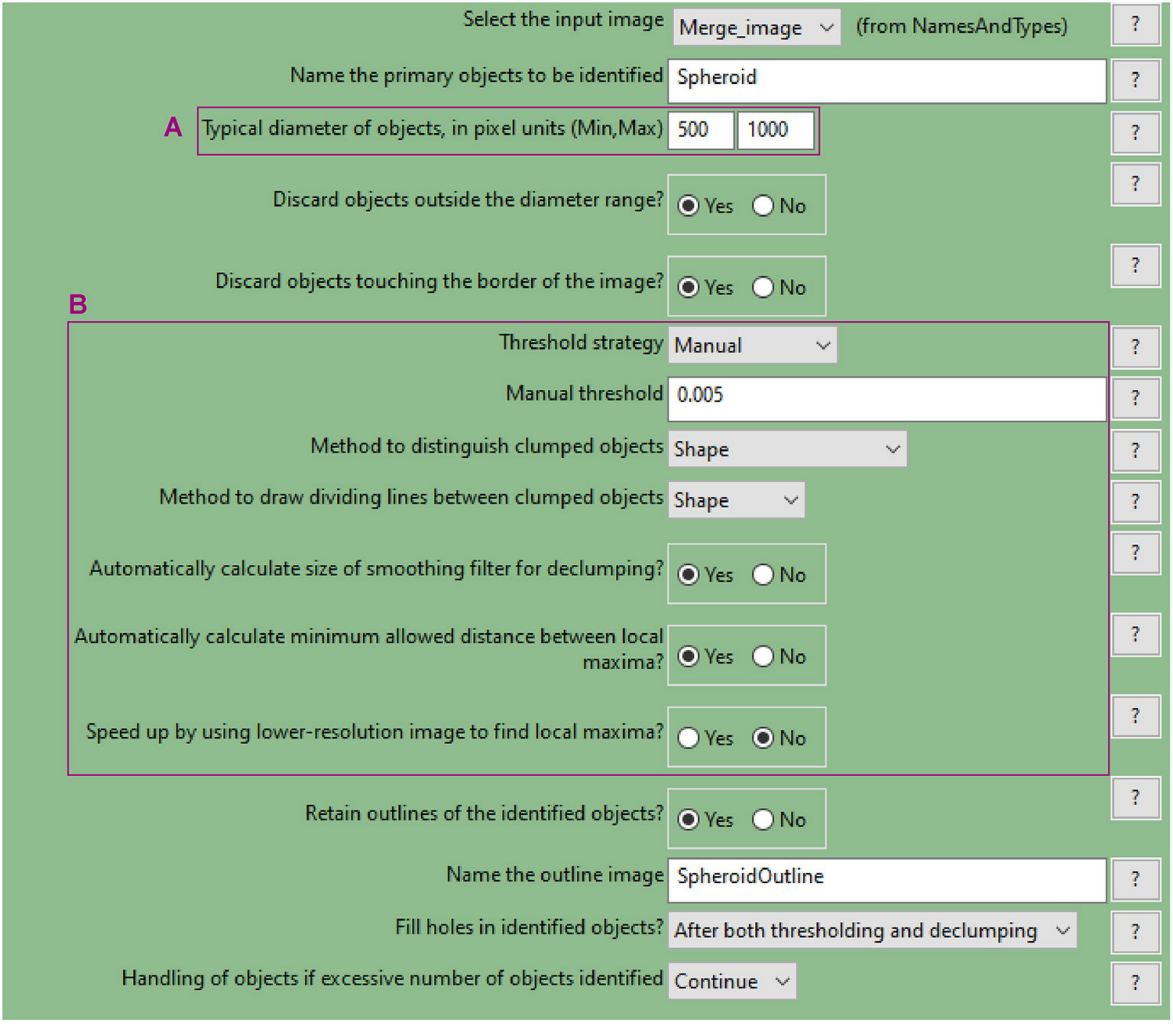
CellProfiler “IdentifyPrimaryObjects” module. Parameters used to detect spheroid outline for analysis shown in Figure 3.

- Similar to step a., use the nuclei image (DAPI image) as input
- Indicate an estimate of the spheroid diameter (Figure 9A) and choose an appropriate threshold strategy (Figure 9B). These parameters will help to identify the spheroid instead of the cells as the object of interest. Manual over automatic threshold is preferred for this task. If necessary, fine tune the remaining parameters.
- Use the test module to optimise accurate detection.

**e. Measure distance of cells from the spheroid surface (“RelateObjects” module)**

**Figure 10:**
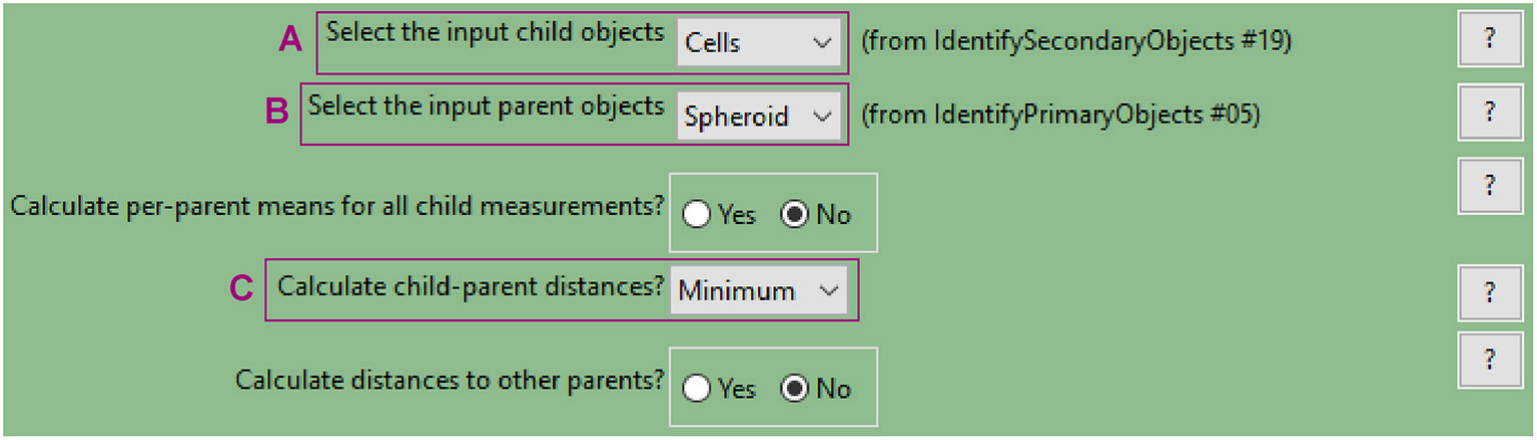
CellProfiler “RelateObject” module. Parameters used to measure cell-to-spheroid-surface distance for analysis shown in Figure 3.

- This module allows for associating child with parent objects. The relevant measurement for this example analysis is the distance of each child object (nuclei or cells, to be defined in the parameters, Figure 10A) to their parent object (spheroid, Figure 10B).
- The minimum distance option (Figure 10C) will measure the distance from the centroid of the child objects (nuclei) to the closest perimeter point on the parent object (spheroid).

**f. Additional modules**

- Modules such as “ClassifyObjects,” “OverlayOutlines,” and “SaveImages” can be combined and added after steps of objects identification and feature measurements to generate images for visual analysis and illustration purposes (Figures 3B,D–G).

**g. “ExportToSpreadsheet” module**

- This module will convert the measurements to a comma-, tab-, or other character-delimited text format and save them. These data are used for further analysis, such as data plotting and statistical analysis.

##### CellProfiler Analysis of DRAQ7™Fluorescence Signal

**Figure 11:**
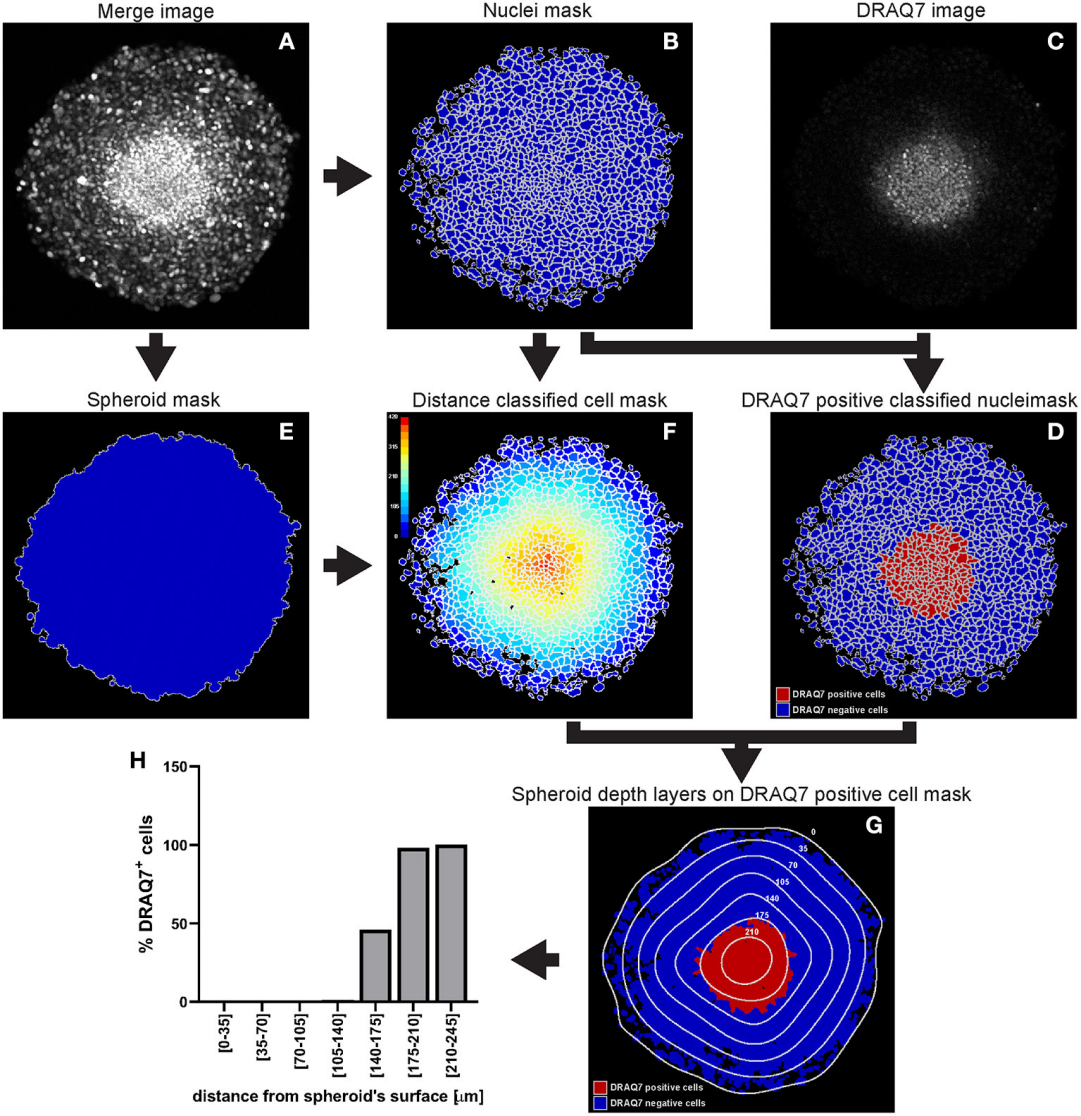
Main steps of CellProfiler DRAQ7 image analysis. The Merge image **(A)** is used to identify the nuclei as primary objects **(B)** which together with the DRAQ7 image **(C)** are exploited to quantify DRAQ7 fluorescent signal for each nucleus and identify DRAQ7 positive nuclei **(D)**. The Merge image **(A)** is also used to determine the spheroid outline **(E)** which is in turn employed to measure the distance of each nucleus from the spheroid surface **(F)**. The data obtained from the CellProfiler analysis can be used to generate a distribution of the DRAQ7 positive cells at different depths in the spheroid **(G,H)**. The colour-coded scale **(F)** indicates the distance from the spheroid surface in μm. In **(G)** the numbers labelling the depth curves indicate the distance from the spheroid surface in μm.

A WM983B melanoma spheroid was formed and cultured in medium supplemented with 3 μM DRAQ7™ then fixed and cleared. A central optical section acquired with a confocal microscope is used here as an example to outline this image analysis (Figure 11).

- **Identify the cell nuclei as explained for hypoxia analysis step a**.
  In the absence of a nuclear staining image, other nuclear signals such as that from expressed fluorescent proteins can be used. This is shown in the example illustrated in Figure 11 where mAG (FUCCI sensor), mKO2 (FUCCI sensor) and DRAQ7™ images were merged to generate a comprehensive image of the cell nuclei in the spheroid (Figure 11A). The Fluorescence Ubiquitination-based Cell Cycle Indicator (FUCCI) is a fluorescent reporter used for monitoring cell-cycle progression where mAG (monomeric Azami Green) and mKO2 (monomeric Kusabira Orange 2) fluorescent proteins are expressed in different phases of the cell cycle, i.e., G2/M phase and G1 phase, respectively (19).
- **Measure DRAQ7**™ **fluorescent signal intensity**
  As explained for hypoxia analysis in step c. but using the identified nuclei instead of cells (DRAQ7™ signal is nuclear).
- **Identify the spheroid**
  As explained for hypoxia analysis step d.
- **Measure distance of nuclei from the spheroid surface**
  As explained for hypoxia analysis step e. (using the identified nuclei instead of cells).
- **Other modules**
  Add and combine “ClassifyObjects,” “OverlayOutlines,” and “SaveImages” modules to generate images for visual analysis and illustration purposes (Figures 11B,D–G).
- **Export measurements and save them as a file (“ExportToSpreadsheet” module)**

##### CellProfiler Analysis of Spheroid-Infiltrating Fluorescent PBMCs

**Figure 12:**
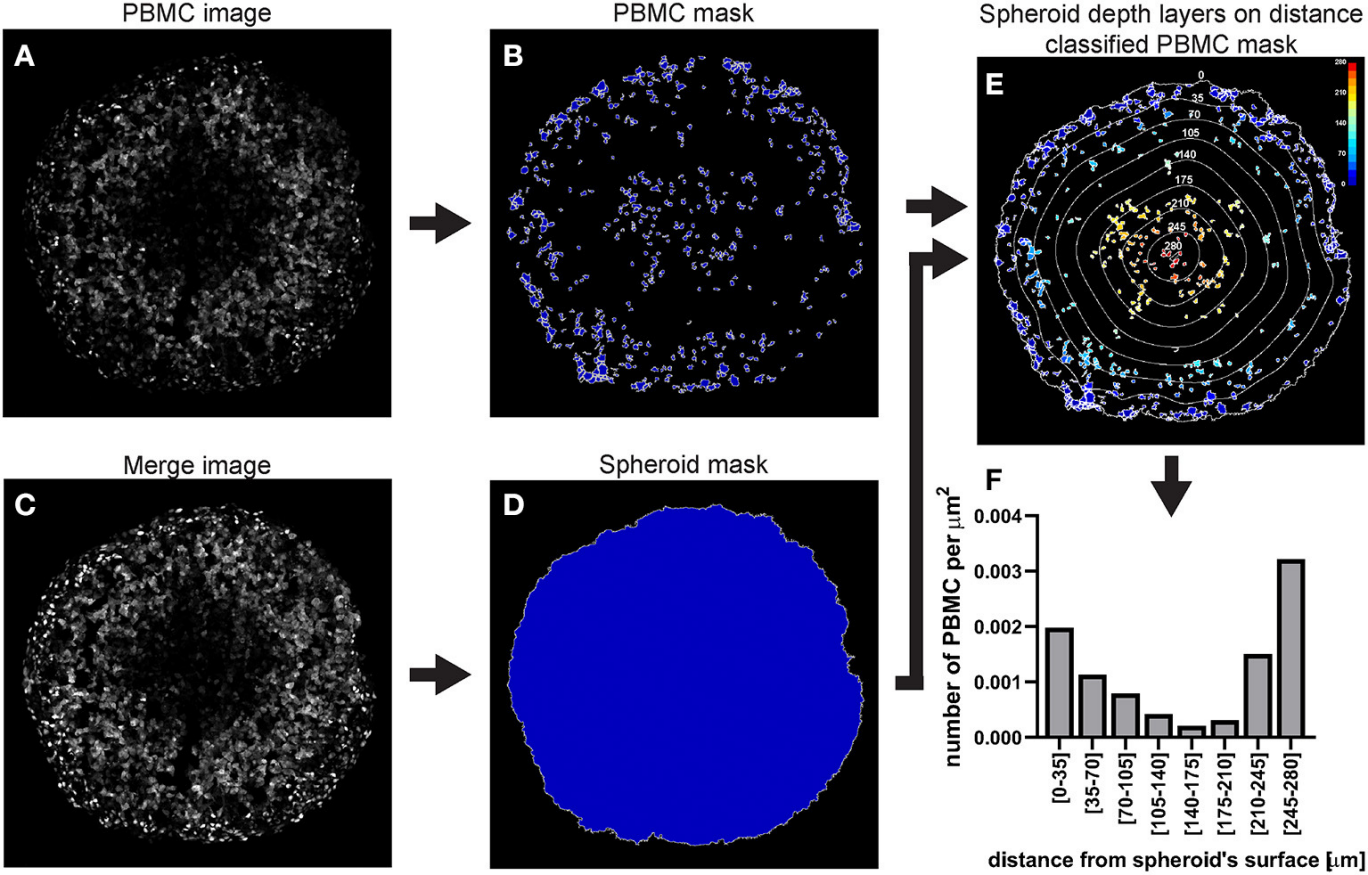
Main steps of CellProfiler PBMC infiltration image analysis. The PBMC image **(A)** is used to identify the PBMC as primary objects **(B)**. The Merge image **(C)** is used to determine the spheroid outline **(D)** which in turn is employed to measure the distance of each PMBC from the spheroid surface **(E)**. The data obtained from the CellProfiler analysis can be used to generate a PBMC infiltration profile at different depths in the spheroid **(F)**. The colour-coded scale **(E)** indicates the distance from the spheroid's surface in μm. In **(E)** the numbers labelling the depth curves indicate the distance from the spheroid's surface in μm.

A C8161 melanoma spheroid was incubated for 24 h with 1 × 10^6^ PBMCs isolated from a healthy individual and fluorescently stained, fixed and cryosectioned. A central cryosection was used as an example to outline this image analysis (Figure 12).

- **Identify the PBMCs using the “IdentifyPrimaryObjects” module**
  As explained for hypoxia analysis step a. but using the PBMC image as input (Figure 12A).
- **Identify the spheroid using the “IdentifyPrimaryObjects” module**
  As explained for hypoxia analysis step d. In the example illustrated in Figure 12 mAG (FUCCI sensor), mKO2 (FUCCI sensor) and PBMC images were merged to generate an image (Figure 12C) that would have allowed identification of the spheroid outline (Figure 12D).
- **Measure the distance of PBMCs from the spheroid surface**
  As explained for hypoxia analysis step e. (using the identified PBMCs instead of cells).
- **Additional modules**
  Add and combine “ClassifyObjects,” “OverlayOutlines,” and “SaveImages” modules to generate images for visual analysis and illustration purposes (Figures 12B,D,E).
- **Export measurements and save them as a file (“ExportToSpreadsheet” module)**

#### Application Example: Detection of Cell Killing by Bortezomib

We provide an example of a C8161 melanoma spheroid treated with bortezomib, a 26S proteasome inhibitor approved for treatment of multiple myeloma and mantel cell lymphoma (20, 21). Bortezomib primarily acts on proliferating melanoma cells *via* a mechanism involving G2/M arrest and apoptosis (22–24), while G1-arrested cells appear to be protected from this cytotoxic effect (25), providing the necessity of cell cycle-tailored targeting of melanoma (26).

In this example a FUCCI-transduced C8161 melanoma spheroid was incubated with 50 nM bortezomib for 48 h and confocal fluorescence images were taken at 0, 24, and 48 h. DRAQ7 was added to the culture medium 24 h prior to bortezomib addition to a final concentration of 3 μM. FUCCI-C8161 spheroids incubated with DRAQ7 can be imaged without clearing to assess cell death. In fact, FUCCI-C8161 spheroids exhibit low light scattering (acceptable image quality) and express the FUCCI cell cycle indicator, while DRAQ7 is a permeable non-toxic compound that becomes fluorescence only when intercalated with double-stranded DNA.

**Figure 13:**
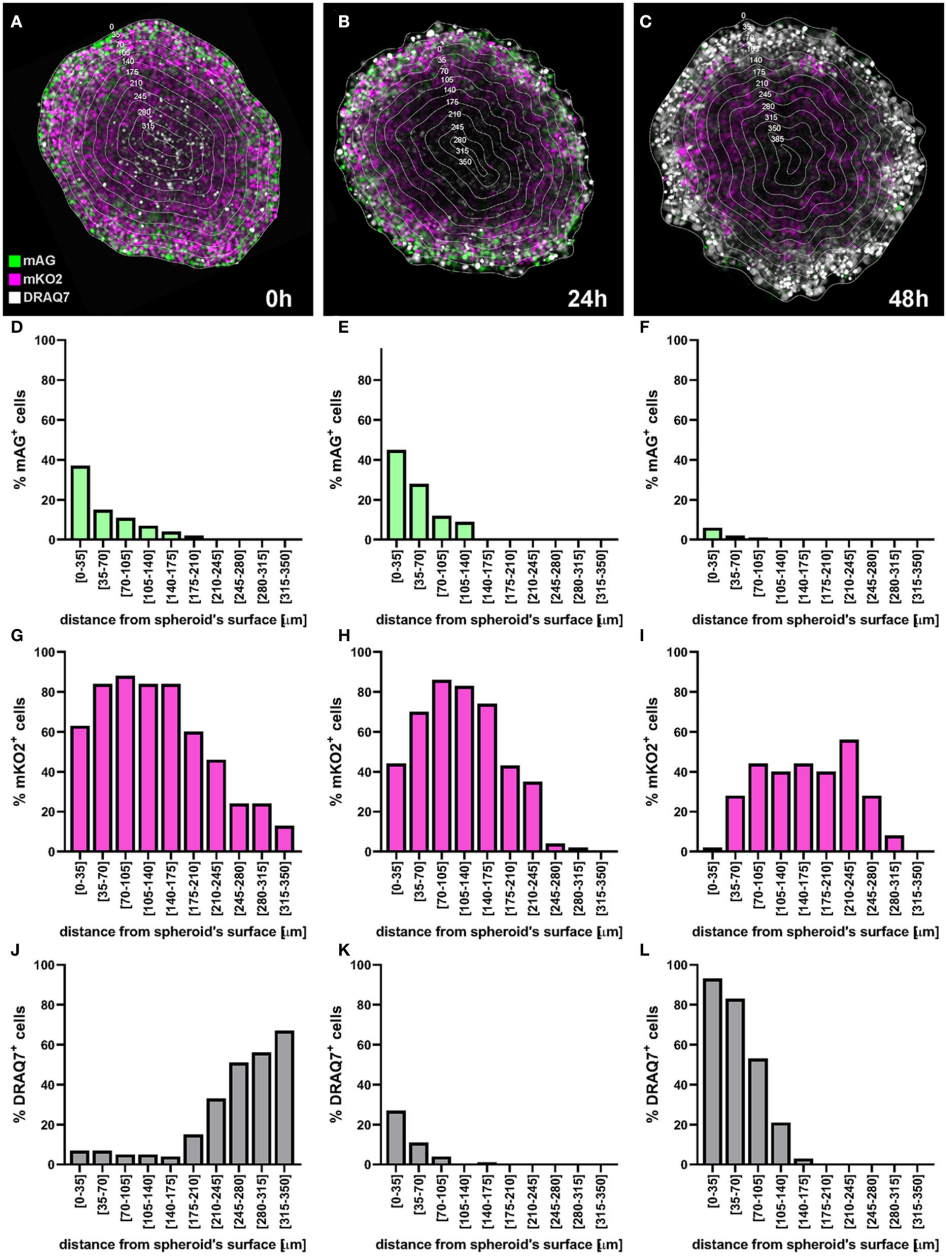
Application example: detection of cell killing by bortezomib. A FUCCI-C8161 melanoma spheroid was incubated with 50 nM bortezomib for 48 h and a central confocal fluorescence image was taken at 0 h **(A)**, 24 h **(B)**, and 48 h **(C)**. DRAQ7 was added to the culture medium 24 h prior to bortezomib addition to a final concentration of 3 μM. Percentages of mAG^+^ **(D–F)**, mKO2^+^ **(G–I)** and DRAQ7^+^ **(J–L)** cells were calculated using an image analysis similar to that applied for the example depicted in Figure 11 and plotted as a function of the distance from the spheroid surface.

At 0 h the signal of the FUCCI fluorescent proteins shows the typical pattern observed in spheroids (Figure 13A), where the percentage of the mAG^+^ cells decreases from the surface toward the centre of the spheroid (Figures 13A,D). This, as explained earlier, is indicative of an actively cycling spheroid periphery and a G1-arrest that increases moving inwards toward the spheroid centre. DRAQ7 fluorescence signal mainly localises in the centre of the spheroids (Figures 13A,J) where a necrotic core lacking FUCCI signal is expected (Figures 13A,D,G). After 24 h an increase in mAG^+^ and DRAQ7^+^ cell percentages is noticeable in the spheroid periphery (Figures 13B,E,K), as expected from the G2-arresting and cytotoxic effect caused by bortezomib on actively cycling cells. Lack of DRAQ7 signal in the spheroid core at this time point is in agreement with our previous observations, whereby while DRAQ7 efficiently marks dying and recently died cells, its fluorescence signal is lost over time by these cells. After 48 h the spheroid periphery shows strong DRAQ7 positivity while mAG^+^ cells are barely present (Figures 13C,F,L), supporting the killing of actively cycling cells by bortezomib. In turn, the inner part of the spheroid shows mKO2 positive cells (Figures 13C,I), which due to their G1-arrested status are expected to be protected from bortezomib G2-arresting and cytotoxic effects.

## Discussion

Inter-patient, intra-patient and intra-tumour genetic and microenvironment-driven heterogeneity makes personalised medicine a crucial requirement for the successful cancer treatment (27). The ability of spheroids to model solid tumours and to mimic multiple aspects of tumour cell biology and microenvironment *in vitro* makes them a useful tool to predict treatment response and could assist in therapy selection for best patient outcome. A major strength of *in vitro* models is that they allow functional, as opposed to only observational, examination.

Here we describe three methods of quantitative and spatial fluorescence analysis of spheroids. Location-dependent data are fundamental because they account for intra-tumoural heterogeneity, a common feature of solid cancers that is regarded as one of the principal causes of therapy resistance.

Spheroids are usually round and symmetrical and so is their intra-spheroid environment-driven heterogeneity (e.g., oxygen concentration presents as a gradient radially decreasing from the surface toward the core). Based on this symmetry, findings obtained from equatorial sections are comprehensive for the whole spheroid as they allow to extrapolate information for any orientation of the spheroid.

However, spatial localisation of different types of cells (e.g., cancer and endothelial cells, fibroblasts, etc.) in spheroid co-cultures may not reflect symmetry, if independent from the microenvironment-driven heterogeneity. In this case, other types of CellProfiler measurements can be used. The “MeasureObjectNeighbors” module determines how many neighbours each object (cells) has, whereby the distance for which objects should be considered neighbours can be specified. This can be applied in the study PBMC infiltration in a fibroblast and melanoma cell co-culture spheroid, where fibroblasts and melanoma cells are not evenly distributed but rather form clusters enriched of one or the other cell type (28). Identifying the number of neighbouring fibroblasts of each cancer cell in the spheroid can describe the cell type distribution, i.e., even or clustered. Applying the same analysis to each infiltrating PBMC, can provide insight into whether PBMCs preferentially localise close to a specific type of cells. Furthermore, in case of asymmetrical spheroids, analysis of spheroid three-dimensional reconstruction is necessary to accurately describe cell localisation. This requires imaging of all optical (confocal microscopy) or physical (cryosections) spheroid sections.

We described here two main spheroid preparations for fluorescence imaging, spheroid cryosectioning and spheroid clearing, each with their own advantages and disadvantages. Spheroid clearing provides a faster and less labour-intense method to achieve high quality spheroid imaging than cryosectioning. However, cryosectioning presents three advantages over spheroid clearing. First, the ability to perform staining without potential limitations of the staining agent diffusion throughout the spheroids, which would vary with sterical and chemical characteristics of the staining agent itself and those of the tumour microenvironment. Second, imaging of cleared spheroids requires confocal microscopes, which may not be as accessible as wide-field fluorescence microscopes. Thirdly, serial sections around the spheroid equator, while not containing the same individual cells, are comparable in terms of spatial heterogeneity and can be used for separate staining and comparison of different features (e.g., overlap of G1-arrested and poorly PBMC-infiltrated area). Imaging of uncleared spheroids *via* confocal microscopy mainly often results in insufficient image quality for CellProfiler analysis, with particularly weak signal from cells located in the spheroid centre. Improved image quality may be achieved with more specialised microscopy equipment such as selective plane illumination microscopy (SPIM). Conclusively, while spheroid clearing is a suitable technique when using highly permeable, small fluorescent compounds (e.g., DRAQ7) and confocal microscopy, spheroid cryosectioning is the optimal method for protocol involving staining with antibodies (e.g., pimonidazole hypoxia detection) and the use of wide-field fluorescence microscopes.

High image quality is of particular importance for software-based quantitative and spatial analysis as it facilitates accurate feature recognition and allows batch processing for high throughput studies. For this we use the open-source image-analysis software CellProfiler because of its approachability as it does not require programming skills and help resources are abundant and easily accessible (detailed manuals, user forums, and pipelines created by other users).

We outline the application of this method for the detection of three important features in anti-cancer therapy assessment: hypoxia, cell death and PBMC infiltration. Low oxygen detection is important as hypoxia presents heterogeneously in virtually all solid cancers, has a strong role in prognosis and therapy response and constitutes a selective target. Cell death measurement lays at the base of anti-cancer drug assessment and verification of CTLs and NK cells infiltration is a pillar to ascertain effective immunotherapy. Nevertheless, the general concepts of this protocol can be applied to any other spheroid feature that can be visualised using microscopy. While fluorescence represents an ideal signal for software-based image analysis, visible light signal could also be exploited if optimum background contrast is achieved.

**Hypoxia detection in patient-derived spheroids** ***via* fluorescent pimonidazole staining** not only could assist in assessing tumour hypoxia status, but also to determine whether cells would be effectively targeted by specific hypoxia-activated prodrugs. Determinants of tumour sensitivity to hypoxia-activated prodrugs are multifactorial and while hypoxia is a necessary prerequisite, it is not sufficient to guarantee drug activation. Currently available biomarkers, such as the presence of specific reducing enzymes, have only partial prediction power. Multiple spheroids generated with the same tumour-derived cells using the methodology outlined here, are expected to be highly comparable in size and shape, and so is the distribution and intensity of their hypoxic zone. Therefore, while hypoxia is examined in a set of untreated spheroids *via* pimonidazole staining, hypoxia-activated prodrugs cytotoxicity can be assessed through DRAQ7™ staining in the other set of spheroids and spatially compared.

**DRAQ7**™ **fluorescent staining of patient-derived spheroids** is a straightforward method to predict cytotoxic drug effects. Compared to general cytotoxicity tests, like LDH assay, it presents the advantage of providing spatial information. This can be pivotal if any response heterogeneity is detected throughout the spheroid. In fact, identification and characterisation of less responsive areas would provide precious insight on alternative potential therapies to combine for best patient outcome.

**Figure 14:**
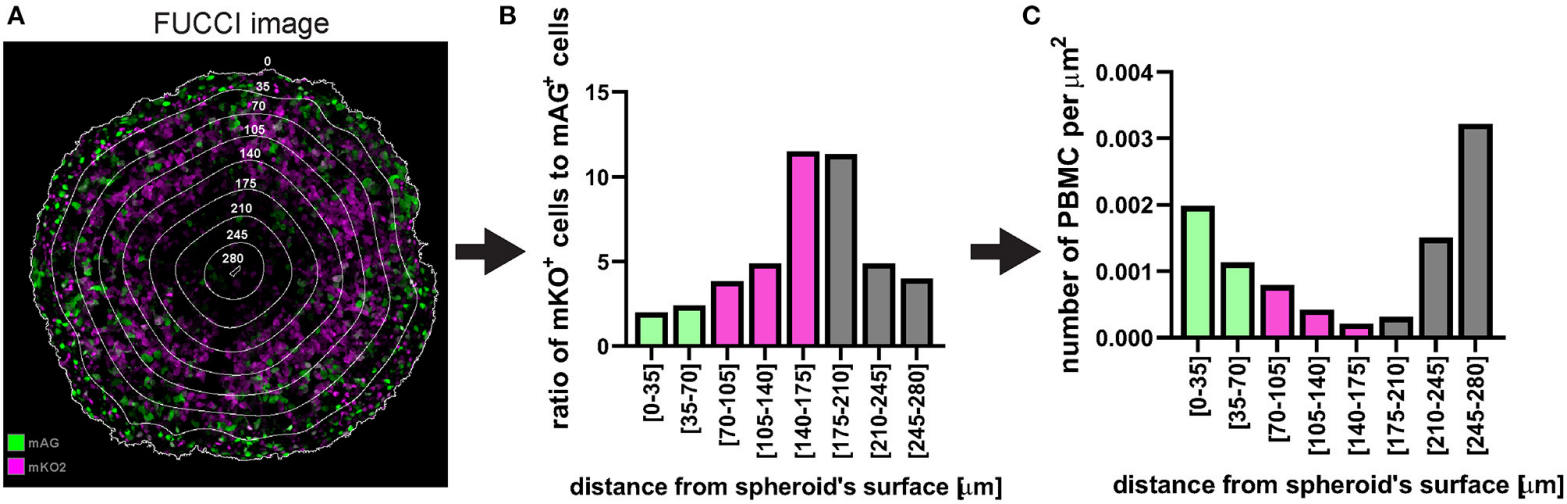
Example of application of fluorescent spheroid-infiltrating PBMC spatial detection analysis. **(A)** FUCCI fluorescence image of the same spheroid section as in Figure 12 with the same distance rings. A mixture of mAG and mKO2 positive cells designates areas of proliferation whereas G1-arrest occurs in zones with increased proportion of mKO2 positive cells. **(B)** Ratio of mKO2 positive cells to mAG positive cells plotted as a function of the distance from the spheroid surface. When this ratio is low it indicates proliferation and when it increases it implies occurrence of G1-arrest. Bar colours were assigned based on visual inspection of **(A)**. Green, proliferation; magenta, G1-arrest; and grey, cell death. Decreased ratio in the necrotic core may be an artefact due to dead cells loosing FUCCI fluorescence. **(C)** Same PBMC infiltration profile as in Figure 12F where bars (spatial bins) have been colour-coded according to the heterogeneous cell populations identified in **(A,B)**.

In the case of **fluorescent spheroid-infiltrating PBMC spatial detection**, the obtained information can be used to generate a profile of the number of infiltrating PBMCs at different depths in the spheroid. In the example used here, the profile of the distance-classified PBMCs (Figure 12), shows that the presence of these cells within the spheroid is moderately frequent in the spheroid periphery, decreases in deeper areas and then increases again in the deepest zone. This information provides insight on the ability of the patient's PBMCs to penetrate and move through a cancer mass with specific chemical and mechanical properties that mimic those of the patient's tumour. While this information is *per se* clinically relevant, comparison of the PBMC infiltration profile with other spatially heterogeneous features of the cancer mass can provide additional insight of preferentially targeted tumour areas. The image in Figure 14A shows Fluorescent Ubiquitinated Cell Cycle indicator [FUCCI (19)] signals (mAG in green for G2/M cells and mKO2 in magenta for G1 cells) acquired from the same spheroid as the one used to obtain the PBMC infiltration profile shown in Figure 12. From this image, we can visually extrapolate that cell proliferation (mixture of mAG positive and mKO2 positive cells) primarily occurs in the spheroid periphery, while the cells are G1-arrested (increased presence of mKO2 positive cells) in deeper zones (10). The absence of FUCCI signal close to the spheroid centre indicates cell death (necrotic core). This is confirmed by the mKO2^+^/mAG^+^ cell ratio at different depths (Figure 14B). Based on the distance bins used to build the PBMC infiltration profile, these three biologically heterogeneous zones fall approximately in the range of 0–70 μm, 70–175 μm, and 175–280 μm from the spheroid surface, respectively. Based on this heterogeneity distribution, it can be extrapolated that PBMCs preferentially infiltrated and accumulated in the proliferating and necrotic zones and less so in the G1-arrested zone (Figure 14C). Spatial correlation between PBMC localisation and cell cycle could be exploited to improve therapy outcome. In this case combination of immunotherapy with a drug targeting G1-arrested cells could achieve a more comprehensive killing of the cells composing the tumour. The example used here illustrates global detection of PBMCs, however additional immunofluorescence staining of spheroid sections can inform on the presence of specific immune cells and cytokines that characterise hot tumours (e.g., CTLs and NKs). Furthermore, spatial comparison and correlation with relevant features for therapy outcome, such as cancer cell killing, are expected to enhance the therapy outcome predictability of this procedure and could be achieved by additional DRAQ7™ staining.

A model is a compromise between reality and feasibility and as such presents some limitations. Considering the enormous impact that immunotherapy is having on cancer treatment, the main caveat of the tool that we have proposed here is perhaps the unavailability of a complete immune system. While this model does not provide a comprehensive immune response as that provided by *in vivo* models, supplementation of patient-derived immune cells delivers a number of important players and aspects that are targeted by immunotherapy. Similar limitations apply to any therapy that exploits features that are not included in this system, such as antiangiogenic agents, due to the lack of vasculature, or drugs targeting stromal cells, e.g., antibodies directed against CAFs expressing prolyl endopeptidase FAP. More complex spheroid models including these additional components could be used for assessment of these therapies but may be more time- and resource-consuming.

In conclusion, we describe a protocol where spheroids are used as fast, inexpensive and reproducible *in vitro* tumour model to quantitatively and spatially examine biological events that are important for cancer prognosis and therapy outcome. We outline this procedure for tumour hypoxia, cell death and immune cell infiltration. Detection of these processes *via* fluorescence microscopy in spheroid cryosections produces excellent image quality for accurate and automated analysis employing user-friendly, open-source Fiji-ImageJ and CellProfiler software. We propose an explorative application of these techniques to assist personalised medicine based on functional assessment and therefore independently form biomarkers.

## Data Availability Statement

The original contributions presented in the study are included in the article/supplementary material, further inquiries can be directed to the corresponding authors.

## Ethics Statement

The studies involving human participants were reviewed and approved by The University of Queensland Human Research Ethics Committees (HRECs). The patients/participants provided their written informed consent to participate in this study.

## Author Contributions

NH: conceptualisation, supervision, project administration, and funding acquisition. LS, GG, and NH: methodology, investigation, validation, formal analysis, data curation, writing—review, and editing. LS and NH: writing—original draft. All authors contributed to the article and approved the submitted version.

## Conflict of Interest

The authors declare that the research was conducted in the absence of any commercial or financial relationships that could be construed as a potential conflict of interest.
